# Supplementary material for: Trpv4-mediated apoptosis of Leydig cells induced by high temperature regulates sperm development and motility in zebrafish
Source: Commun Biol. 2024 Jan 13;7:96. doi: 10.1038/s42003-023-05740-y (PMC10787748; doi:10.1038/s42003-023-05740-y)
Supplement: Supplementary file 2 — Supplementary Information [file 42003_2023_5740_MOESM2_ESM.pdf]

Supplementary Table 1

name	sequence	application	Related Figure
zebrafish *β-actin* qPCR forward	CTCTTCCAGCCTTCCTTCCT	qRT-PCR	Figure 1B, Figure 2A, Figure 3B Figure 4G and Supplementary Figure 7
zebrafish *β-actin* qPCR reverese	CACCGATCCAGACGGAGTAT	qRT-PCR	Figure 1B, Figure 2A, Figure 3B Figure 4G and Supplementary Figure 7
zebrafish *Hsp70* qPCR forward	ATCCTCATGGGCGACACCTCTG	qRT-PCR	Figure 1B and Supplementary Figure 7
zebrafish *Hsp70* qPCR reverese	TGTCGGCCTCTTGCACCATTCTC	qRT-PCR	Figure 1B and Supplementary Figure 7
zebra *Hspa5*-1 qPCR forward	ATCAGATCTGGCCAAAATGC	qRT-PCR	Figure 1B and Supplementary Figure 7
zebra *Hspa5*- 1 qPCR reverese	CCACGTATGACGGAGTGATG	qRT-PCR	Figure 1B and Supplementary Figure 7
zebra *Hspa5*-2 qPCR forward	CCAGAGCCAAGTTTGAGGAG	qRT-PCR	Figure 1B and Supplementary Figure 7
zebra *Hspa5*-2 qPCR reverese	TCGGAAGCAGTGGAGAAGAT	qRT-PCR	Figure 1B and Supplementary Figure 7
zebra *Hsp90*-1 qPCR forward	GGCTTCTACTCCGCTTTCCT	qRT-PCR	Figure 1B and Supplementary Figure 7
zebra H*sp90*-1 qPCR reverse	TCTACGGTCTCGGTCTTGCT	qRT-PCR	Figure 1B and Supplementary Figure 7
zebra H*sp90*-2 qPCR forward	GTTCGACGAGAGCGATAAGG	qRT-PCR	Figure 1B and Supplementary Figure 7
zebra *Hsp90*-2 qPCR reverse	CCTGTCTGGTAAGCCTGAGC	qRT-PCR	Figure 1B and Supplementary Figure 7
zebrafish *Star* qPCR forward	CGGGGAGAAGGTCATGAGTA	qRT-PCR	Figure 1B and Figure 3B
zebrafish *Star* qPCR reverse	ACAGGTGGGTCCATTCTCAG	qRT-PCR	Figure 1B and Figure 3B
zebrafish *Insl3* qPCR forward	TGAAGCTTTGTGGTCGTGAG	qRT-PCR	Figure 1B and Figure 3B
zebrafish *Insl3* qPCR reverse	TCCCTCCACTGCTCATCTTC	qRT-PCR	Figure 1B and Figure 3B
zebrafish *Cyp11c1* qPCR forward	GCTGATCAGAGCCAACATCA	qRT-PCR	Figure 1B and Figure 3B
zebrafish *Cyp11c1* qPCR reverse	CGGTGATTCCCACAGGATAC	qRT-PCR	Figure 1B and Figure 3B
zebrafish *Cyp17a1* qPCR forward	TGGAGCTCTTTGCATGTTTG	qRT-PCR	Figure 1B and Figure 3B
zebrafish *Cyp17a1* qPCR reverse	GATTCCCTGGCTGTACTGGA	qRT-PCR	Figure 1B and Figure 3B
zebrafish *Hsd3β* qPCR forward	GATCCGACTGCTGGATAGAAACA	qRT-PCR	Figure 1B and Figure 3B
zebrafish *Hsd3β* qPCR reverse	CCCGGCAATCATCAAGAGA	qRT-PCR	Figure 1B and Figure 3B
zebrafish *Piwil1* qPCR forward	ATACCGCTGCTGGAAAAAGG	qRT-PCR	Figure 1B and Supplementary Figure 7
zebrafish *Piwil1* qPCR reverse	GCAAGACACACTTGGAGAACC	qRT-PCR	Figure 1B and Supplementary Figure 7
zebrafish *Dazl* qPCR forward	ACTGGGACCTGCAATCATGA	qRT-PCR	Figure 1B and Supplementary Figure 7
zebrafish *Dazl* qPCR reverse	AATACAGGTGATGGTGGGGC	qRT-PCR	Figure 1B and Supplementary Figure 7
zebrafish *Sycp3* qPCR forward	AGAAGCTGACCCAAGATCATTCC	qRT-PCR	Figure 1B and Supplementary Figure 7
zebrafish *Sycp3* qPCR reverse	AGCTTCAGTTGCTGGCGAAA	qRT-PCR	Figure 1B and Supplementary Figure 7
zebrafish *Odf3b* qPCR forward	GATGCCTGGAGACATGACCAA	qRT-PCR	Figure 1B and Supplementary Figure 7
zebrafish *Odf3b* qPCR reverse	CAAAGGAGAAGCTGGGAGCTT	qRT-PCR	Figure 1B and Supplementary Figure 7
zebrafish *Sox9a* qPCR forward	GAGGAAGTCGGTGAAGAACG	qRT-PCR	Figure 1B and Supplementary Figure 7
zebrafish *Sox9a* qPCR reverse	TCTCGTTTCAGATCCGCTTT	qRT-PCR	Figure 1B and Supplementary Figure 7
zebrafish *Amh* qPCR forward	TAACCCTCACGTTCCCAAAG	qRT-PCR	Figure 1B and Supplementary Figure 7
zebrafish *Amh* qPCR reverse	TAAGGTGAATGTGGCCATGA	qRT-PCR	Figure 1B and Supplementary Figure 7
zebrafish *Dmrt1* qPCR forward	GGAGGAGATGGGCATCTGTA	qRT-PCR	Figure 1B and Supplementary Figure 7
zebrafish *Dmrt1* qPCR reverse	GTAATAGGAGGCGTCCACCA	qRT-PCR	Figure 1B and Supplementary Figure 7
zebrafish *Insl3* in situ forward	TGAAGCTTTGTGGTCGTGAG	*in situ* hybridization	Figure 1C , Figure 2C and Figure 3C
zebrafish *Insl3* in situ reverese	TGAGTCCACAAATGCTACCTG	*in situ* hybridization	Figure 1C , Figure 2C and Figure 3C
zebra *Hspa5* in situ forward	ATCAGATCTGGCCAAAATGC	*in situ* hybridization	Figure 1D and Figure 3D
zebra *Hspa5* in situ reverese	GAAATCTCCACCGGAGTGAA	*in situ* hybridization	Figure 1D and Figure 3D
zebrafish *Trpv1* qPCR forward	GGCTACCTGCTTCTGACAGG	qRT-PCR	Figure 2A
zebrafish *Trpv1* qPCR reverese	CTTGTACGAGGGCTTCTTGC	qRT-PCR	Figure 2A
zebrafish *Trpv4* qPCR forward	GAGCGACGATGCAAACAGTA	qRT-PCR	Figure 2A
zebrafish *Trpv4* qPCR reverese	GTGAGAGGTGTCGAGCTTCC	qRT-PCR	Figure 2A
zebrafish *Trpm4a* qPCR forward	GGAGATGAGGATGGGTCAGA	qRT-PCR	Figure 2A
zebrafish *Trpm4a* qPCR reverese	AGCGACATTTCTCTGCGATT	qRT-PCR	Figure 2A
zebrafish *Trpm4b1* qPCR forward	TTTATTGCTGGCTTGTGCTG	qRT-PCR	Figure 2A
zebrafish *Trpm4b1* qPCR reverese	TATTTGTCGCTGTGCTCCTG	qRT-PCR	Figure 2A
zebrafish *Trpm4b2* qPCR forward	GGATAGAGCCGTGCAGAGAC	qRT-PCR	Figure 2A
zebrafish *Trpm4b2* qPCR reverese	TCAATGCTGGCTGAAGTGAC	qRT-PCR	Figure 2A
zebrafish *Trpm4b3* qPCR forward	CTCTGGGCGATAACTTCAGC	qRT-PCR	Figure 2A
zebrafish *Trpm4b3* qPCR reverese	CCTCCAGACAGGCTCTGAAC	qRT-PCR	Figure 2A
zebrafish *Trpm5* qPCR forward	GGCTAAACTGGAGCATGAGC	qRT-PCR	Figure 2A
zebrafish *Trpm5* qPCR reverese	TTGATCCATTAAGGCGGAAG	qRT-PCR	Figure 2A
zebrafish *Trpv4* in situ forward	GGGGTTCCTAACCCTATGGA	*in situ* hybridization	Figure 2B and 2C
zebrafish *Trpv4* in situ reverese	TGATGATGCCCAGATTTTGA	*in situ* hybridization	Figure 2B and 2C
zebrafish *20β-hsd* forward	TGCACGAGTGGTCAATGTGTC	qRT-PCR	Figure 4G
zebrafish *20β-hsd* reverese	ACTAGCTGTCCATGCGGCTCT	qRT-PCR	Figure 4G
zebrafish *20β-hsd* in situ forward	ATATGCGACCAAGGAAGCTG	*in situ* hybridization	Figure 4H
zebrafish *20β-hsd* in situ reverese	TGTGGAAGGAGGATTTGAGG	*in situ* hybridization	Figure 4H
zebrafish *Sycp3* in situ forward	ACTGACGGTGTGGTGACAGA	*in situ* hybridization	Supplementary Figure 4
zebrafish *Sycp3* in situ reverese	CTGCAACAGCTCCAATTGTTA	*in situ* hybridization	Supplementary Figure 4
zebrafish *Odf3b* in situ forward	GGGGCAACTGGAATGAATAA	*in situ* hybridization	Supplementary Figure 4
zebrafish *Odf3b* in situ reverese	TGTTGGAGCGACGTACTCAG	*in situ* hybridization	Supplementary Figure 4
zebrafish *Trpv4* guide RNA	CCTCTAATACGACTCACTATAGGTCATGTGATGAGGGATCCCGTTTAAGAGCTATGC	guide RNA synthesis	Supplementary Figure 5
zebrafish *Trpv4* geno forward	AGCACTCTGTGGAAGCCCTA	genotyping	Supplementary Figure 5
zebrafish *Trpv4* geno reverese	TCCTTGTTGATGTGGCAAAA	genotyping	Supplementary Figure 5
medaka *β-actin* forward	TGGCGCTTGACTCAGGATTT	qRT-PCR	Figure 10C
medaka *β-actin* reverese	GCAGATGCCTGGGGTGTTTA	qRT-PCR	Figure 10C
medaka *Trpv1* qPCR forward	AAGAGCTGCTACGGGACAAA	qRT-PCR	Figure 10C
medaka *Trpv1* qPCR reverese	GCTGCCTCAAACAAGAGGTC	qRT-PCR	Figure 10C
medaka *Trpv4* qPCR forward	GAAGAAACGTCGCAAAAAGC	qRT-PCR	Figure 10C
medaka *Trpv4* qPCR reverese	CCGGTGGATGGTTCTCTAAA	qRT-PCR	Figure 10C
medaka *Trpm2* qPCR forward	CCTGAACACCTGACAGAGCA	qRT-PCR	Figure 10C
medaka *Trpm2* qPCR reverese	CTGAAATAGCCTGGGAGCAG	qRT-PCR	Figure 10C
medaka *Trpm4* qPCR forward	TGTCCTTCTCCTCGTCGTCT	qRT-PCR	Figure 10C
medaka *Trpm4* qPCR reverese	GCAAAGTCCTGGCTCTTGAC	qRT-PCR	Figure 10C
medaka *Trpm5* qPCR forward	GGTCGGTGTCAGAGAAGAGC	qRT-PCR	Figure 10C
medaka *Trpm5* qPCR reverese	TTGGCCACCTCAAAGAGAGT	qRT-PCR	Figure 10C
medaka *Trpa1*-1 qPCR forward	ACGTCACCCTGATCCAGTTC	qRT-PCR	Figure 10C
medaka *Trpa1*-1 qPCR reverese	GCCAAGTGTAAAGGGGACAA	qRT-PCR	Figure 10C
medaka *Trpa1*-2 qPCR forward	CTCCATGAAGCTGTGCAAAA	qRT-PCR	Figure 10C
medaka *Trpa1*-2 qPCR reverese	ACACAGCAGTCCAGCAGATG	qRT-PCR	Figure 10C
medaka *Hsp70* chr.7 qPCR forward	CTGCCACCTTCTCTCTTTGG	qRT-PCR	Figure 10C
medaka *Hsp70* chr.7 qPCR reverese	ACGGCATTCTAAACGTGTCC	qRT-PCR	Figure 10C
medaka *Hsp70* chr.13 qPCR forward	ATGTCACCCCCTTGTCTCTG	qRT-PCR	Figure 10C
medaka *Hsp70* chr.13 qPCR reverese	ACACGTTCATGATGCCGTTA	qRT-PCR	Figure 10C
medaka *Hsp70* chr.16 qPCR forward	CACGAAACAAACCCAGACCT	qRT-PCR	Figure 10C
medaka *Hsp70* chr.16 qPCR reverese	TCCTTGCTCAGTCGTCCTTT	qRT-PCR	Figure 10C

Supplementary Figure 1

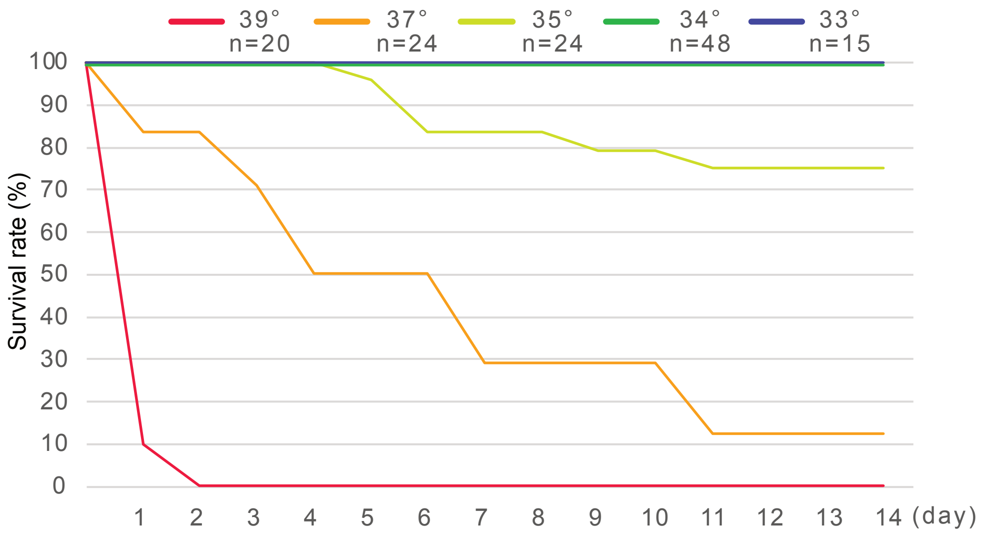

Supplementary Figure 1: Survival rates of adult zebrafish over two weeks of incubation at various temperature.

Supplementary Figure 2

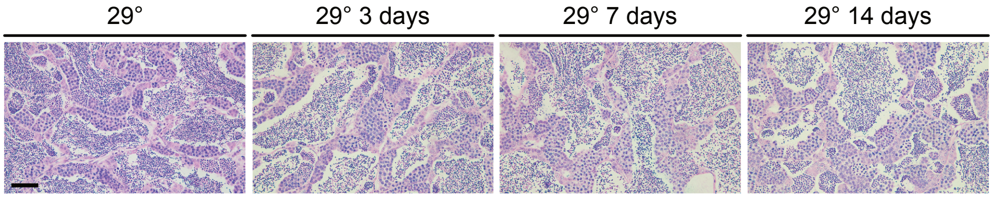

Supplementary Figure 2: No abnormalities in spermatogenesis and changes in testicular morphology were observed when reared at 29° in the temperature stimulation system. Scale bars are 50 μm.

Supplementary Figure 3

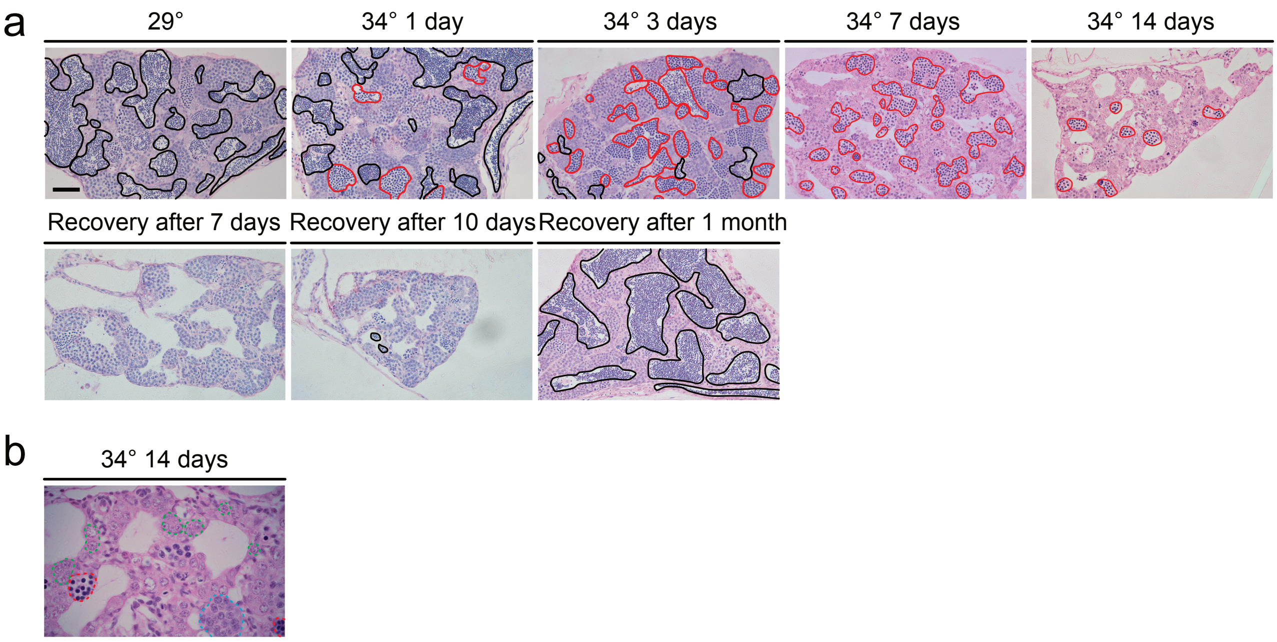

Supplementary Figure 3: Temperature stimulation led to interstitial tissue loss and impaired spermatogenesis. (a) Low-magnification representation of HE staining images shown in Figure 1A. The black line indicates E3 spermatid. The red line indicates an abnormal cell population. (b) There were no morphological abnormalities observed in type A and type B stem cells in testes treated at 34°C temperature for 2 weeks. Green dotted line indicates type A spermatogonia. Blue dotted line indicates type B spermatogonia. Red dotted line indicates the abnormal cell population. Scale bars are 50 µm.

Supplementary Figure 4

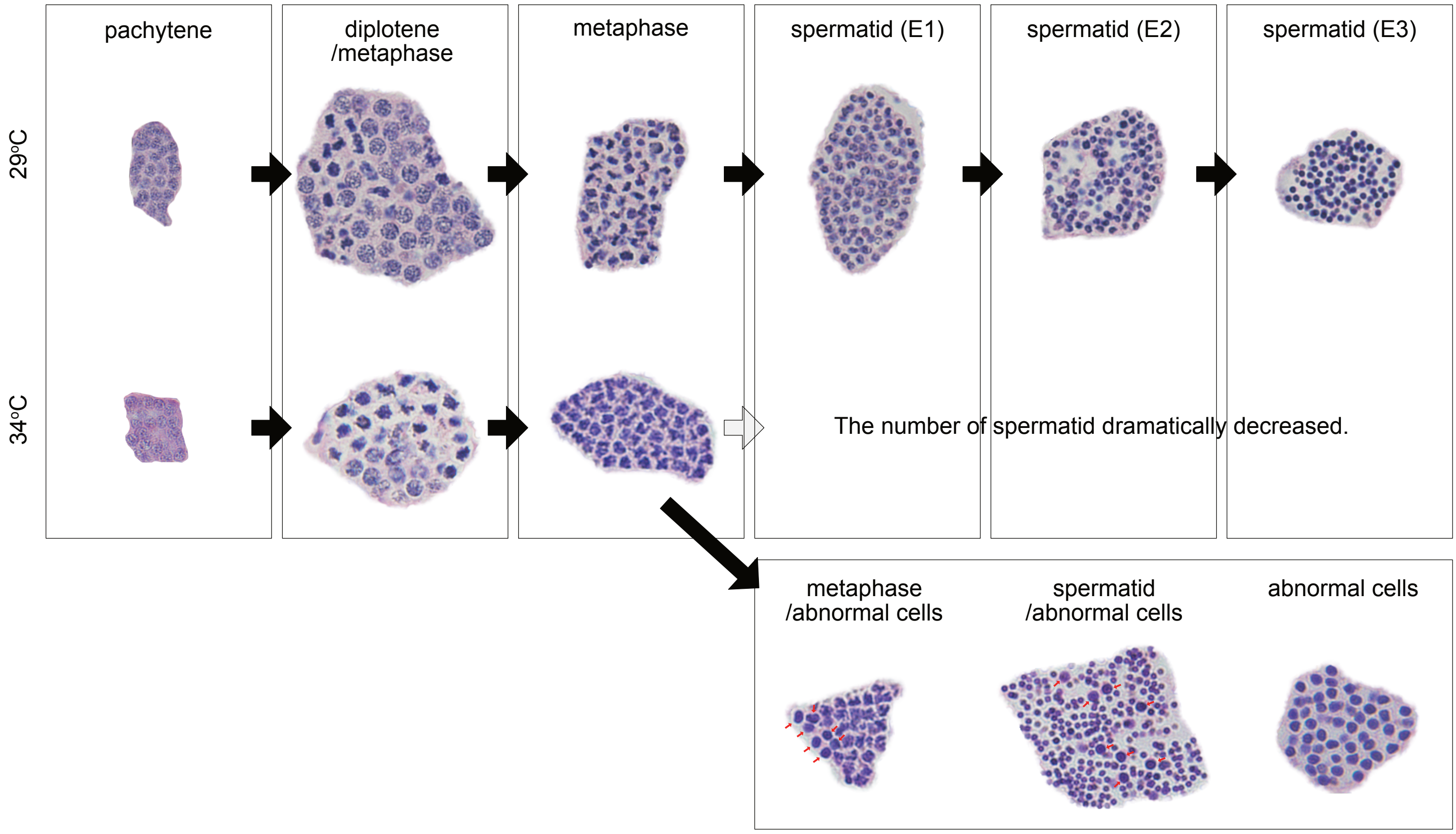

Supplementary Figure 4: Temperature stimulation interferes with normal differentiation from metaphase to spermatid. Each image represents a typical developmental stage of germ cells within one cyst. At 29°C, germ cells differentiated normally into E3 spermatids. At 34°C, normal morphology was observed up to metaphase, but the number of spermatids decreased and abnormal cells were observed in the testes. While cysts with a mixture of normal and abnormal cells were observed with short duration of temperature stimulation, cysts containing only abnormal cells were observed with longer incubation of 34°C. Red arrows indicate abnormal cells.

Supplementary Figure 5

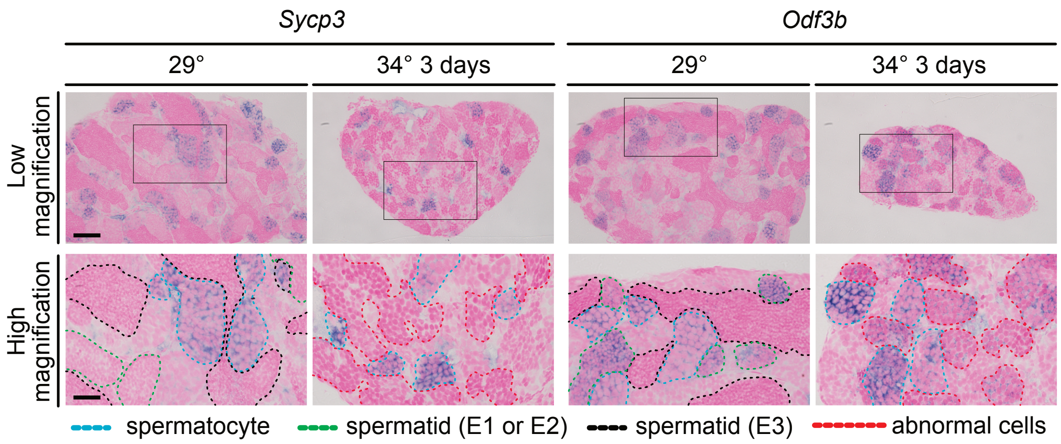

Supplementary Figure 5: Abnormal cells observed after high temperature showed spermatid-like gene expression. *Sycp3* is a marker of spermatocytes (indicated by blue dotted-line). *Odf3b* is normally expressed in spermatocytes and early spermatids (indicated by green dotted-line). In the abnormal cell group (red dotted-line), *Sycp3* was not expressed, while *Odf3b* was expressed. The inset is magnified. Scale bars are 50 μm (Upper) and 20 μm (Lower).

Supplementary Figure 6

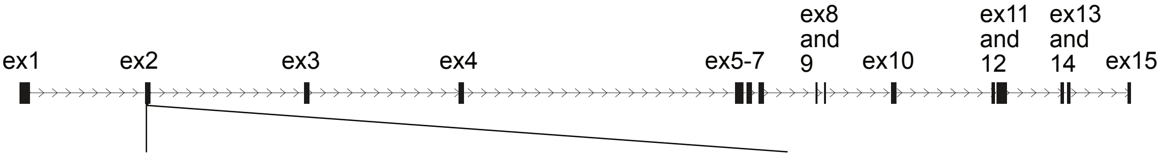


```
WT        GGGATGTCATGTGATGAGGGATCCCCGGAGCCCCCTGTT
ex2-Δ8    GGGATGTCATGTGATGAGGGA -------- GCCCCCTGTT
```

```
WT        GGGATGTCATGTGATGAGGGATCCCCGGAGCCCCCTGTTCTCAAAGTCTTCAACCGCTGG
           G  M  S  C  D  E  G  S  P  E  P  P  V  L  K  V  F  N  R  W

ex2-Δ8    GGGATGTCATGTGATGAGGGAGCCCCCTGTTCTCAAAGTCTTCAACCGCTGGATGTTGTT
           G  M  S  C  D  E  G  A  P  C  S  Q  S  L  Q  P  L  D  V  V

WT        ATGTTGTTCGAAGCTGTATCTCGAGCAGATCCAAGAGCTCTGGACGGCCTTCTGCAGTATCTT
           M  L  F  E  A  V  S  R  A  D  P  R  A  L  D  G  L  L  Q  Y  L

ex2-Δ8    CGAAGCTGTATCTCGAGCAGATCCAAGAGCTCTGGACGGCCTTCTGCAGTATCTTCAGTCCCA
           R  S  C  I  S  S  R  S  K  S  S  G  R  P  S  A  V  S  S  V  P

WT        CAGTCCCACGAAAAGAGGCTGACCGATGAAGAGTTCAAAGAA
           Q  S  H  E  K  R  L  T  D  E  E  F  K  E

ex2-Δ8    CGAAAAGAGGCTGACCGATGA
           R  K  E  A  D  R  *
```

Supplementary Figure 6: Design of Crispr-cas9 targeting *Trpv4*. The guide RNA was designed against a sequence in the 2nd exon of *Trpv4*, resulting in an 8 bp deletion. Predicted amino-acid sequences of ex2 Δ8 is shown. Asterisk indicates the stop codon.

Supplementary Figure 7

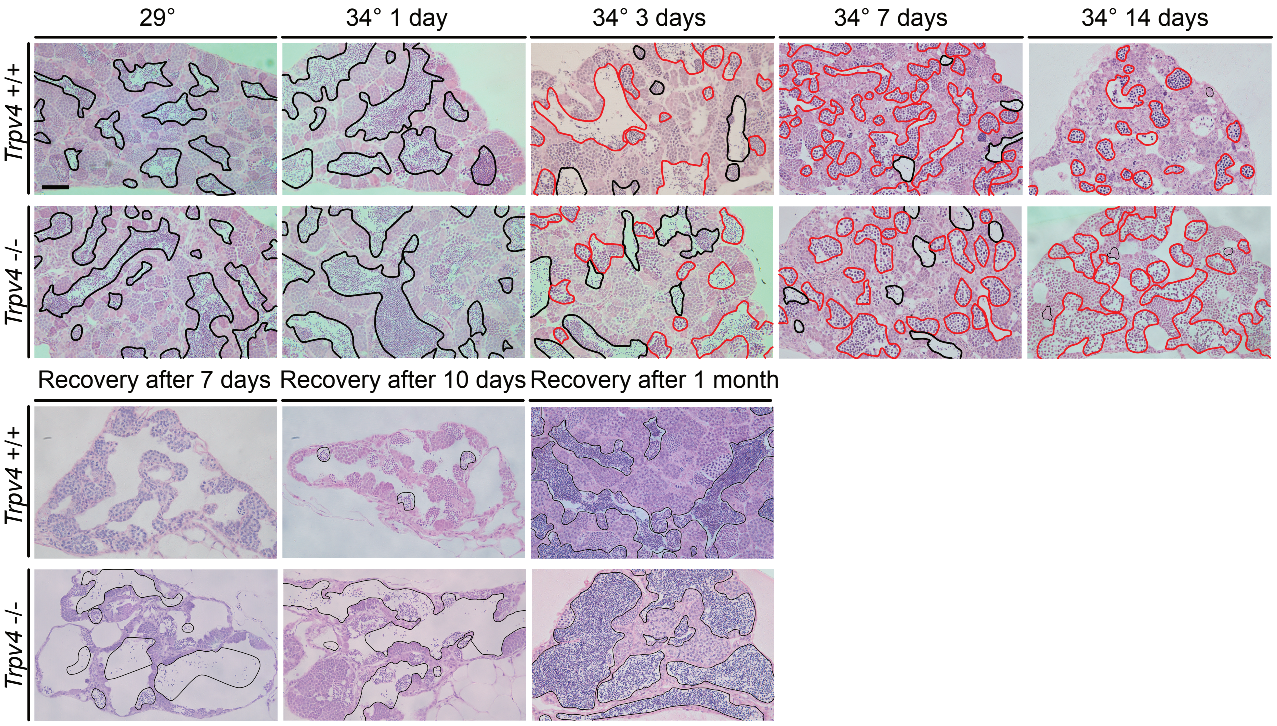

Supplementary Figure 7: Interstitial tissue of *Trpv4* $^{-/-}$ was affected less by high temperature. Low-magnification representation of HE staining images shown in Figure 3A are displayed. The black dotted line indicates E3 spermatid. The red dotted line indicates an abnormal cell population. Scale bars are 50 µm.

Supplementary Figure 8

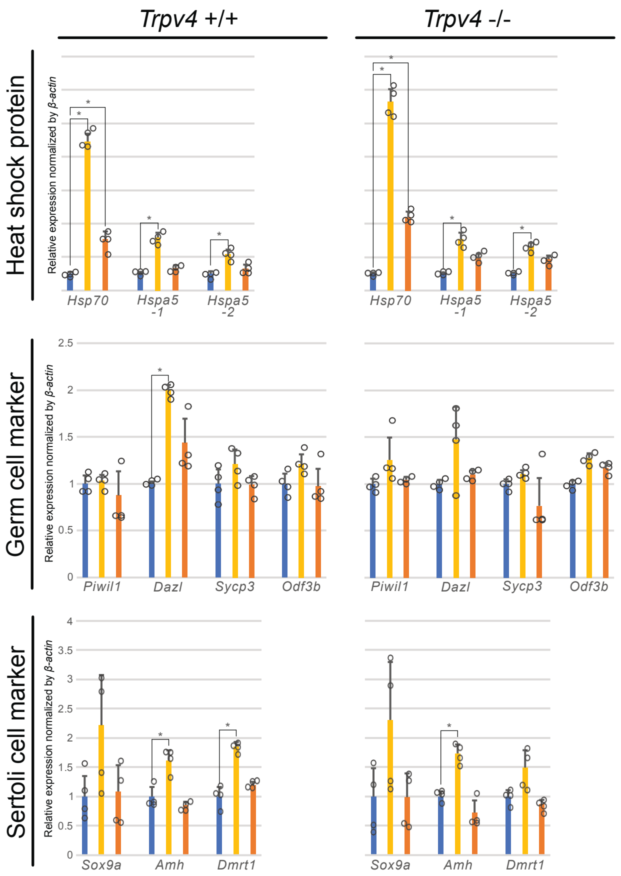

Supplementary Figure 8: High temperature stimulation led to upregulated expression of *Hsp* in testes. qRT-PCR analysis of *Hsp* genes and marker genes for germ cells and sertoli cells marker. Values are the mean ± SEM (n=4). * $p<0.01$ compared with the 29 °C testis.

Supplementary Figure 9

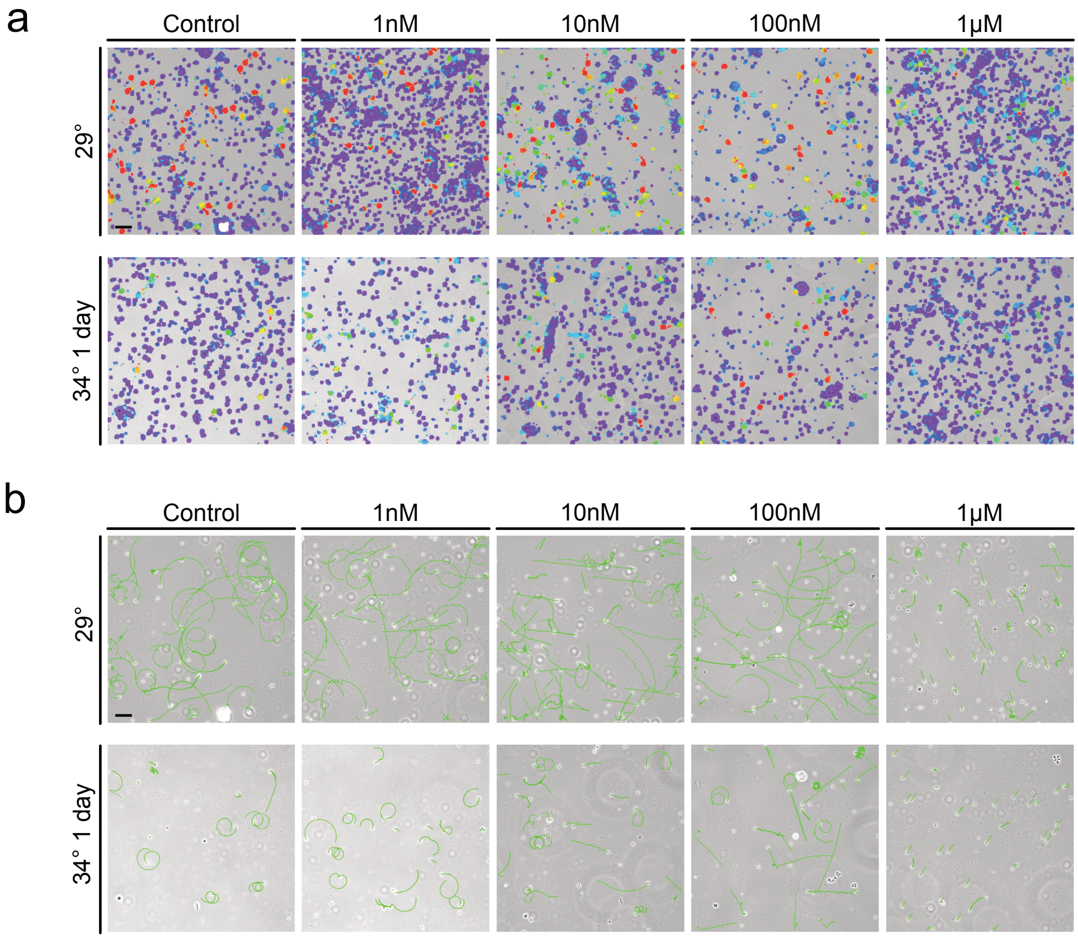

Supplementary Figure 9: Effects of 20β-S exposure on sperm motility. (a) Heat map images of sperm motility in 20β-S. Red indicates high motility and light blue indicates low motility. (b) Tracking of sperm swimming in 20β-S. Scale bars are 50 µm.

Supplementary Figure 10

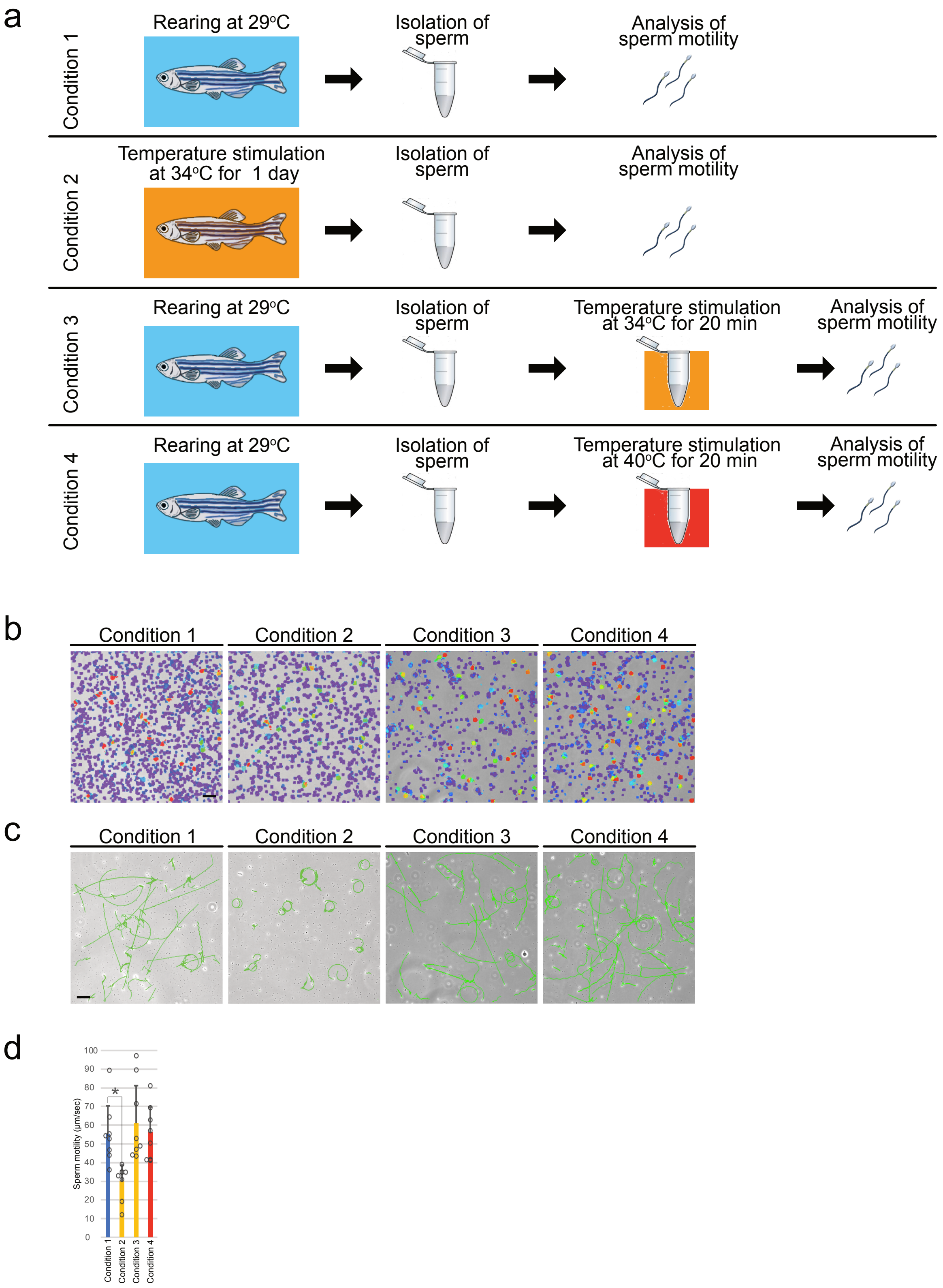

Supplementary Figure 10: Endocrine regulation of the sperm motility by Leydig cells. (a) Experimental paradigm of sperm motility analysis. (b) Heat map images of sperm motility. (c) Tracking of sperm swimming. (d) Averaged sperm motility in four groups. Values are the mean ± SEM (n = 8). * $p<0.01$ compared with the 29°C. Scale bars are 50 µm.

Supplementary Figure 11

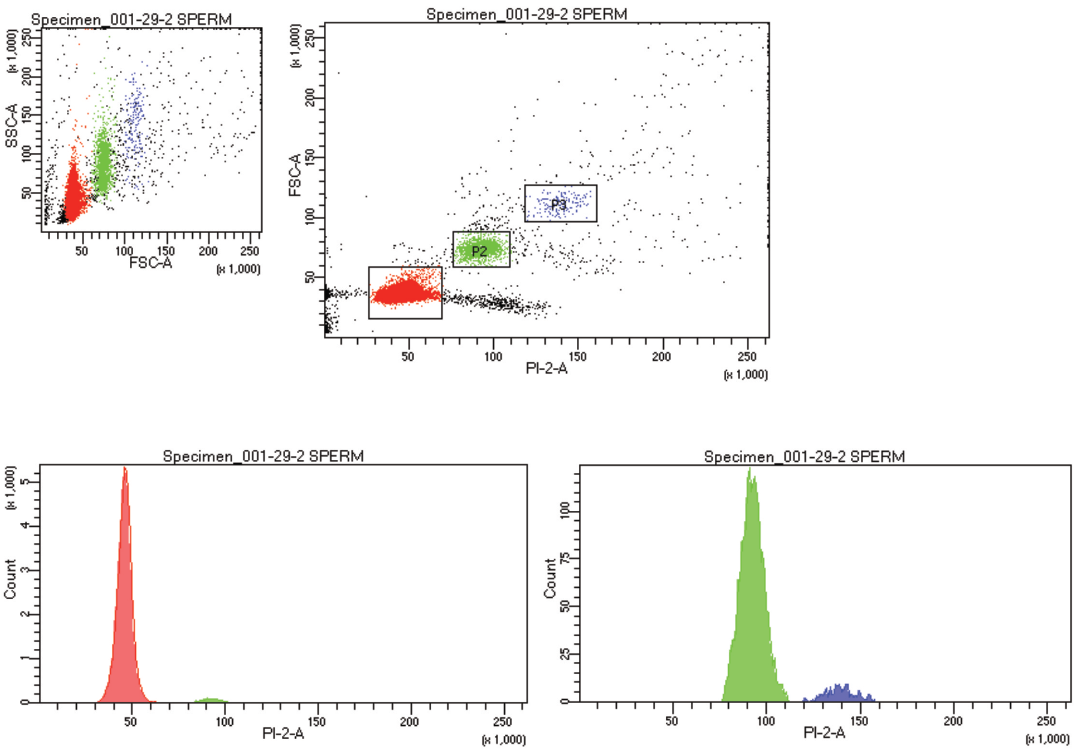

Supplementary Figure 11: Flow cytometry analysis
Cells with smaller cell size and smaller PI signal were designated as mature sperm fractions (red). PI staining allowed separation by nuclear phase (red: n, green: 2n and blue: 4n).

Supplementary Figure 12

		WT	Trpv4 -/-
29°	Leydig function	Normal	Normal
	Sperm motility	Normal	Normal
	Sperm quality	Normal	Normal
34° 1 day	Leydig function	Abnormal (• Apoptosis • *20β-hsd* expression ↓)	Normal
	Sperm motility	Abnormal • Swimming velocity ↓ ⇦ 20β-S exposure improved motility	Normal • Normal swimming velocity
		Abnormal • Fertilization rate ↓↓	Abnormal • Fertilization rate ↓
	Sperm quality	Abnormal (• Abnormal spermatogenesis • Abnormal development in the next generation ↑)	Abnormal (• Abnormal spermatogenesis • Abnormal development in the next generation ↑)

Supplementary Figure 12: Summary of results. Note our proposal the fertilization depend on both sperm motility and sperm quality.

Supplementary Figure 13

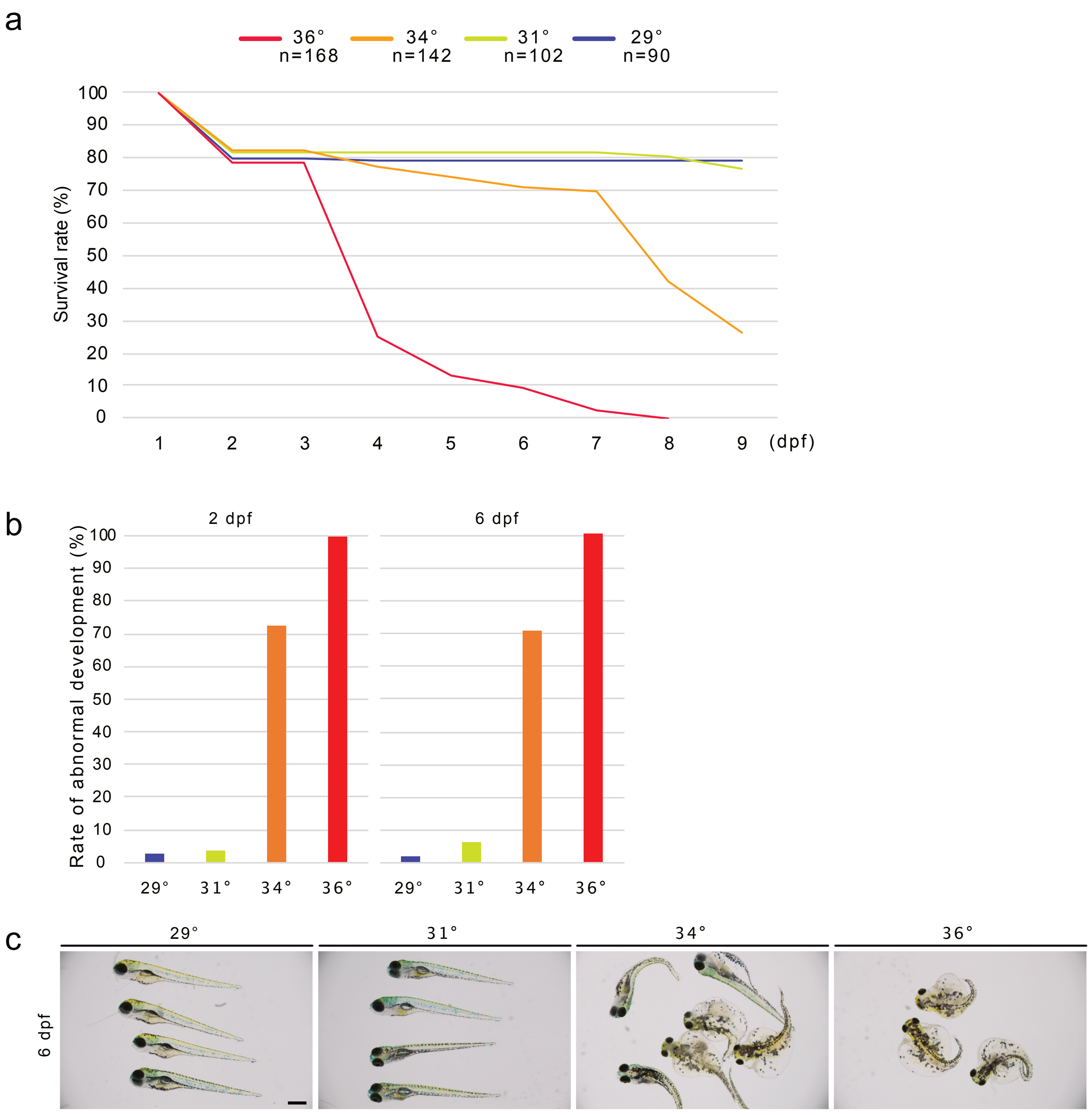

Supplementary Figure 13: High temperature environment led to anomalies in early zebrafish development. (a) Survival rates of zebrafish embryos up to 9 days of incubation at various temperatures. (b) Rates of abnormal development at 2 dpf and 6 dpf embryos. (c) Images of embryo at 6 dpf. Scale bar was 500 µm.

Supplementary Figure 14

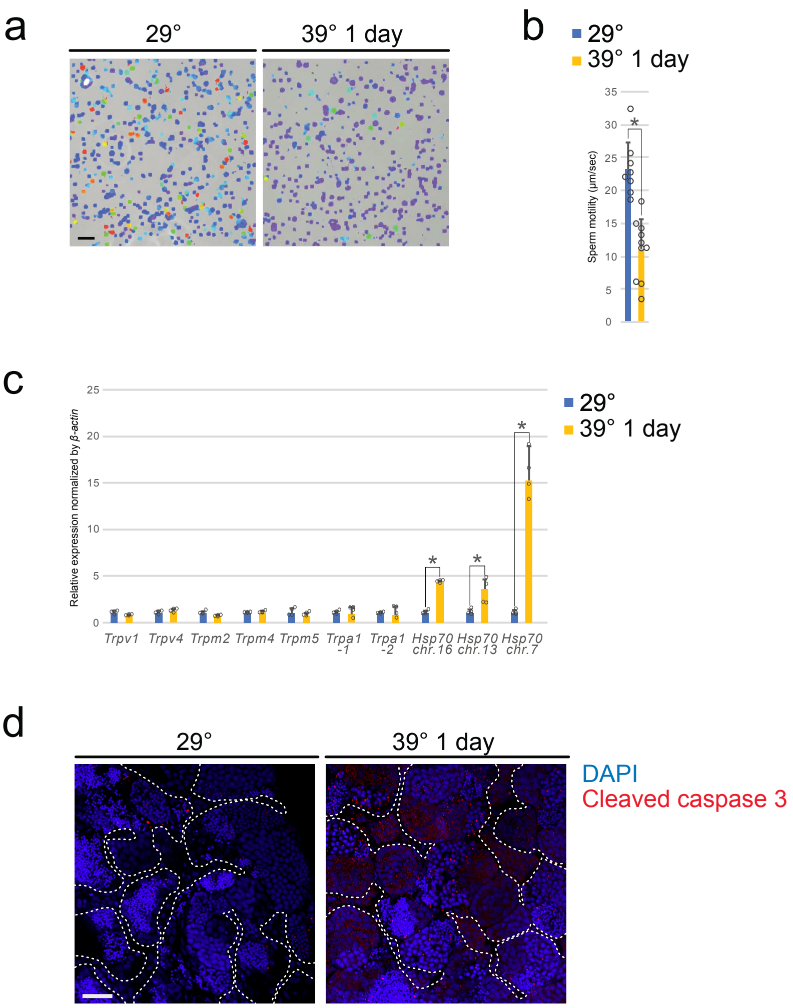

Supplementary Figure 14: *Trp*-mediated apoptosis of Leydig cells not induced in medaka. (a) Heat map images of sperm motility. Red indicates high motility and light blue indicates low motility. (b) Averaged of sperm motility. Values are the mean ± SEM (n=8). * p<0.01 compared with the 29 °C. (c) qRT-PCR analysis of *Trp* family genes in the testis. Values are the mean ± SEM (n=4). * p<0.01 compared with the 29 °C testis. (d) Localization of cleaved caspase 3. White dotted-line indicates interstitial cells. Scale bars are 20 μm.
