# Supplementary material for: Trpv4-mediated apoptosis of Leydig cells induced by high temperature regulates sperm development and motility in zebrafish
Source: Commun Biol. 2024 Jan 13;7:96. doi: 10.1038/s42003-023-05740-y (PMC10787748; doi:10.1038/s42003-023-05740-y)
Supplement: Supplementary file 3 — Description of Additional Supplementary Files [file 42003_2023_5740_MOESM3_ESM.pdf]

Description of Additional Supplementary Files

File name: Supplementary Data 1
Description: The source data behind the figures.

File name: Supplementary Movie 1
Description: Trpv4 +/+, sperm motility at 29°C.

File name: Supplementary Movie 2
Description: Trpv4 +/+, sperm motility at 34°C.

File name: Supplementary Movie 3
Description: Trpv4 -/-, sperm motility at 29°C.

File name: Supplementary Movie 4
Description: Trpv4 -/-, sperm motility at 34°C.
